# Time-dependent cortical responses to siesta disruption in male mice

**DOI:** 10.3389/fnins.2025.1613747

**Published:** 2025-08-22

**Authors:** Jingwei Ding, Shigenobu Shibata, Tatsuhiko Kubo, Yu Tahara

**Affiliations:** ^1^Graduate School of Biomedical and Health Sciences, Hiroshima University, Hiroshima, Japan; ^2^Laboratory of Physiology and Pharmacology, School of Advanced Science and Engineering, Waseda University, Tokyo, Japan

**Keywords:** siesta disruption, circadian timing, stress response, frontal cortex, transcriptomics

## Abstract

**Introduction:**

Siestas, or daytime naps, play a critical role in relieving sleep pressure and maintaining physiological balance. However, the effects of siesta disruption remain largely unexplored.

**Methods:**

In this study, we disrupted the natural siesta period (ZT20–23) through daily bedding changes for 2 weeks and examined its effects on overall stress levels, sleep architecture, behavior, and transcriptional responses in the frontal cortex.

**Results:**

Siesta disruption during the late dark phase led to increased core body temperature, locomotor activity, and wakefulness, while having minimal effects on subsequent light phase sleep patterns, behavioral performance, or serum stress markers. Transcriptomic analysis at ZT0 and ZT12 revealed distinct time dependent responses: ZT0 was associated with the activation of stress-related and homeostatic pathways, whereas ZT12 showed enrichment of genes related to neuronal structure and intracellular transport.

**Discussion:**

These findings suggest that even short-term siesta disruption induces mild but phase-specific cortical adaptations, involving early stress mitigation at ZT0 followed by synaptic remodeling at ZT12.

## 1 Introduction

Daytime naps or siestas alleviate sleepiness, enhance cognitive performance, and promote emotional well-being (Boukhris et al., 2020; Leong et al., 2021; Hsouna et al., 2020; Cremone et al., 2017). Additionally, siestas exert stress-releasing and immune-boosting effects by reversing salivary interleukin-6 and urinary norepinephrine changes induced by sleep restriction (Faraut et al., 2015). Siestas can also decrease oxidative stress associated with sleep deprivation and intense physical exercise in athletes, both of which are substantial sources of physiological stress (Romdhani et al., 2020; Romdhani et al., 2022). Furthermore, siestas have been linked to reduced risk of Alzheimer’s disease (Asada et al., 2000).

Despite epidemiological findings and human experiments, few animal studies have investigated the mechanisms and effects of siestas. In nocturnal rodents, a consistent rest period during the second half of the wake phase has been termed a “siesta” in prior studies (Collins et al., 2020; Ehlen et al., 2015; Whitney et al., 2016). This term is used here to denote a defined mid-active-phase rest episode. Collins et al. (2020) identified a siesta centered around ZT20 (zeitgeber time [ZT] refers to the hours after light onset, where lights on is ZT0 and lights off is ZT12) based on activity data, and Ehlen et al. (2015) observed a rest period peaking at ZT21 using electroencephalography (EEG)/Electromyography (EMG) data. Collins et al. (2020) further demonstrated that a specific population of vasoactive intestinal polypeptide neurons in the suprachiasmatic nucleus is active during siesta, when most suprachiasmatic nucleus neurons are silent. They found that these neurons are necessary and sufficient to regulate the timing of siestas without affecting light-phase sleep. Ehlen et al. (2015) showed that Ube3a^m−/p+^ mice, an Angelman syndrome model, have a reduced capacity to accumulate sleep pressure and do not take siestas, suggesting that siestas are governed by sleep pressure and that Ube3a is a key regulator of sleep homeostasis. Whitney et al. (2016) reported that serotonin deficiency eliminates the siesta period in mice, suggesting that serotonin is essential for siestas. These studies have provided valuable insights into the biological mechanisms underlying siesta behavior, yet the physiological consequences of siesta disruption have received limited attention in animal research.

A recent study in macaques showed that a 30-min nap improved behavioral performance and cognitive function by desynchronizing cortical activity and promoting neural network reorganization (Kharas et al., 2024). However, the molecular and physiological functions of siestas remain underexplored, especially under disruption conditions. To address this gap, we disrupted the natural siesta period (ZT20–23) in mice by performing daily bedding changes—a method previously used to suppress light-phase sleep without affecting peripheral circadian clocks (Tahara et al., 2015). We investigated the effects of siesta disruption on behavior, sleep architecture, and systemic stress levels, with a particular focus on the frontal cortex—a brain region especially vulnerable to sleep loss (Leenaars et al., 2012; Verweij et al., 2014). To examine time-dependent effects, we analyzed both cortical and systemic responses at ZT0 (immediately after siesta disruption) and ZT12 (following the subsequent light-phase sleep, 12 h after siesta disruption).

## 2 Materials and methods

### 2.1 Animals and intervention

All experiments were approved by the Committee for Animal Experimentation of Hiroshima University (approval no. A23-115-2). The experiments were performed in accordance with the law (No. 105) and notification (No. 6) of the Japanese government.

Male ICR mice (8 weeks old; Charles River Laboratories Japan, Inc.) were housed individually under controlled environmental conditions: room temperature 23 ± 2°C, humidity 40% ± 10%. Following a one-week acclimation period under a 12:12 light–dark cycle (lights on at 08:00 and off at 20:00), the light schedule was shifted to 22:00–10:00 to accommodate the experimental interventions. The mice were acclimated to new light cycles for 3 weeks until their activity patterns were fully adapted. All mice were provided ad libitum access to food and water. n = 4–5 mice per group at each time point.

In our mouse model, we observed reduced activity and body temperature during natural siesta (approximately ZT20–23). Mice without any intervention served as the control group. For siesta disruption model establishment, beddings were replaced twice daily at ZT20 and ZT22 using beddings previously utilized by mice within the same group. To prevent the mice from reusing the original bedding, fresh bedding was introduced every 5 days. A single bedding change typically increased activity for about 1 hour; however, a change at ZT20 alone was insufficient to fully suppress the siesta, as rest behavior resumed around ZT22–23 (Supplementary Figure S1).

Compared to other commonly used sleep disruption methods—such as gentle handling or auditory stimulation—bedding change offers a more practical alternative for siesta-specific interventions. Manual stimulation (e.g., tapping or brushing) is labor-intensive and subject to variability between experimenters, while auditory stimuli may not reflect the natural context of siesta interruption. Bedding change, by contrast, passively increases spontaneous activity may mimic real-world behaviors such as post-meal walking in humans to counteract drowsiness. Moreover, this method is well suited for repeated interventions over extended periods.

**Figure 1:**
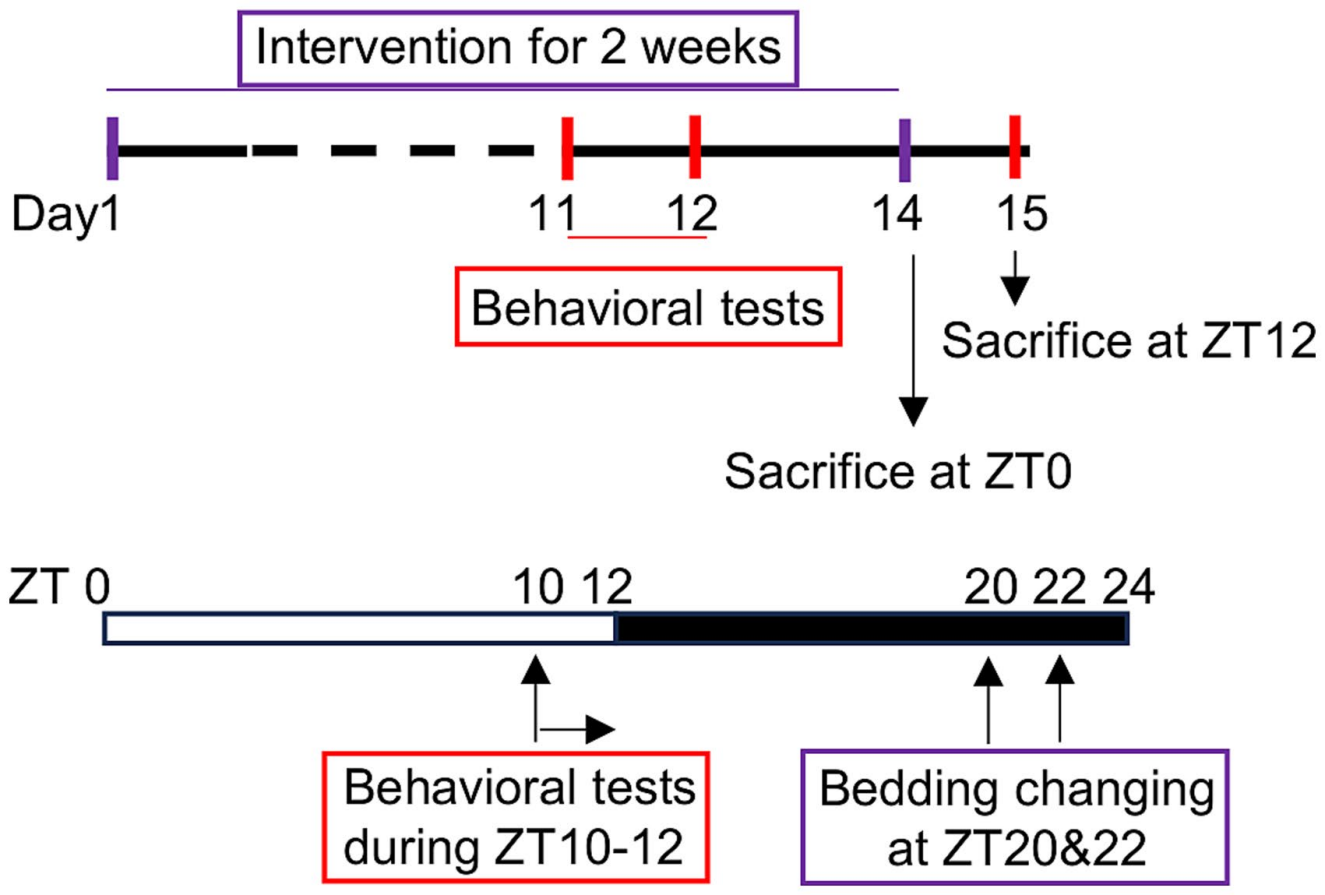
Schematic overview of the siesta disruption experiment. Under baseline conditions, mice exhibit a natural siesta period during the dark phase, typically between ZT20 and ZT23. Mice in the control group were left undisturbed, while those in the siesta disruption group underwent bedding changes at ZT20 and ZT22 daily for 2 weeks to disrupt siesta. Behavioral testing was conducted during ZT10–12 on Day 11 and 12. Animals were sacrificed at either ZT0 or ZT12 for physiological and molecular analyses.

Body weight and food intake were monitored weekly throughout the study. On Day 14, immediately following the final siesta disruption, 5 mice in each group were sacrificed at ZT0 to capture the immediate molecular responses. The remaining mice were sacrificed at ZT12 on Day 15, 12 hours after the final siesta disruption. This timing followed a full light-phase sleep opportunity, enabling the assessment of delayed effects after partial recovery from siesta disruption. Serum and frontal cortex samples were collected for subsequent analyses. A schematic of the experimental timeline is shown in Figure 1.

### 2.2 Serum preparation and quantitative assays performed with kits

Blood was collected from mice and allowed to clot at room temperature for over 30 min. The samples were then centrifuged at 3,000 × g for 20 min at 25°C. The serum was carefully collected and stored at −80°C until further analyses. Serum corticosterone (501320) (Cayman Chemical, Ann Arbor, MI), superoxide dismutase activity (SOD, 706002) (Cayman Chemical, Ann Arbor, MI), and Malondialdehyde (MDA, M496) (Dojindo, Kumamoto, Japan) were measured using ELISA kit. Total antioxidant capacity (TAC) was assessed using a ready to use kit (AC01D) (Metallogenics, Chiba, Japan).

### 2.3 Measurement of locomotor activity

Spontaneous locomotor activity was individually monitored using an infrared radiation sensor (SE-10; Akizuki Denshi Tsusho Co., Ltd., Tokyo, Japan) in accordance with our previously described method (Tahara et al., 2024). Movements were detected and recorded in 20-min epochs. Data collected for 2 weeks were averaged and analyzed using CLOCKLAB software (Actimetrics, IL, USA).

### 2.4 Measurement of core body temperature

Core body temperature was measured using a small temperature sensor (Thermochron SL; KN Laboratories Inc., Osaka, Japan) designed to resemble a button battery. The mice were anesthetized with isoflurane, and the sensor was implanted with a sensing surface positioned near the liver. After a recovery and light shift period of 3 weeks, temperature recordings were automatically taken at 20-min intervals for 10 days. Data were analyzed using RhManager software (Version 2.27; KN Laboratories Inc., Osaka, Japan) as reported previously (Ohnishi et al., 2014).

### 2.5 Sleep measurement with EEG and EMG recording

Surgical implantation of a sleep-monitoring head mount (Pinnacle Technology, Inc., USA) was performed under isoflurane anesthesia. Four stainless-steel EEG electrodes (#8209 0.10″EEG Screws, Pinnacle Technology, Inc., USA) were implanted into the skull over the cortex to monitor EEG signals. Silver conductive epoxy adhesive (MG Chemicals, Canada) was applied to the screws and contact surfaces of the head mount to ensure electrical conductivity. The head mount was securely affixed to the skull using resin cement (Super-Bond, Sun Medical, Japan). Two pre-soldered stainless-steel wires for EMG recording were inserted into the neck muscles. After securing the head mount, an ultraviolet-cured adhesive (Transbond, 3 M Japan Innovation, Japan) was used to stabilize the mount and seal the surgical incision.

Following a one-week recovery period, the animals underwent at least 2 days of habituation in the recording cages before the sleep recordings commenced. The measurements were conducted for two consecutive days under two conditions: control and siesta disruption. EEG and EMG signals were amplified and filtered (0.75–30 Hz) using a Data Conditioning and Acquisition System connected to preamplifiers and digitized through SIRENIA® software (Pinnacle Technology, Inc., USA). The digitized data were analyzed semi-automatically using SleepSign® software (Kissei Comtec, Japan) and visually inspected for accuracy. Sleep stages were classified as follows: wakefulness (Wake), high EMG activity; non-rapid eye movement (NREM) sleep, low EMG activity and a prominent delta band power (0.5–4 Hz); and rapid eye movement (REM) sleep, minimal EMG activity and a prominent theta band power (7–8 Hz). For additional analysis, EEG data were subjected to fast Fourier transformation within the 0–24.2 Hz range using SleepSign®. The hourly theta/delta power density (%) during REM and NREM sleep was calculated as the ratio of the theta/delta band power to the total EEG power within the same frequency range for each hour of light-phase sleep.

### 2.6 Behavioral tests

All behavioral tests were conducted at the end of the light phase (ZT10–12) on day 11 or day 12. All sessions were recorded using a video camera, and the videos were analyzed by a trained observer blinded to the group assignments. Initially, each group consisted of 10 animals used for general experimentation; however, not all behavioral tasks were performed in this primary cohort. To assess additional behavioral outcomes, including the open field test, object location test, and novel object recognition test, a follow-up cohort of 5 animals per group was used. Consequently, sample sizes varied (*n* = 5–10 per group) depending on the specific behavioral assay.

#### 2.6.1 Open field test

The open field test was conducted to assess anxiety-like behavior. One day before testing, each mouse was acclimatized to the testing arena (30 cm × 44 cm, made of white polyvinyl chloride) for 10 min. On the testing day, the mice were individually placed in the center of the arena and allowed to explore freely for 10 min. The arena floor was divided into quadrants, and movements were tracked for the first 5 min. The time spent in the center and the number of entries into the center were recorded. After each test, the arena was cleaned with 70% ethanol to remove the olfactory cues before testing the next animal.

#### 2.6.2 Object location test and novel object recognition test

The object location test was conducted a day after the open field test, following a standard protocol (Denninger et al., 2018), using the same arena as that for the open field test. The mice were exposed to two identical objects affixed to the arena floor using double-sided tape and allowed to explore for 10 min. After a retention interval of more than 30 min, one object was moved to a new position in the arena, whereas the other remained in its original location. The mice were reintroduced into the arena and allowed to explore for 10 min. Subsequently, the novel object recognition test was conducted after another retention interval of more than 30 minutes. During this test, one of the familiar objects from the object location test was replaced with a novel object that differed in shape, size, and color, whereas the other object remained in the familiar location. The mice were placed in the center of the arena and allowed to explore for 10 min. Exploration is defined as the sniffing or touching of an object. The time spent investigating each object was recorded, and the percentage of the total exploration time spent on the novel object or location was calculated as follows: Percentage of total investigation time = (time with a novel location or object) / (time with a novel location or object + time with a familiar location or object) × 100.

#### 2.6.3 Forced swim test

Each mouse was individually placed in a transparent cylindrical tank (20 cm in height, 13 cm in diameter) filled with water maintained at 23–25°C. The test duration was 6 min, and the behavior of the mice was analyzed during the last 4 min. Immobility, defined as floating with minimal movements necessary to keep the nose above the water, was recorded. Struggling behavior, characterized by rapid forelimb movements that caused the front paws to break the water surface, was also measured. In addition, swimming behavior, defined as paddling movements of the forelimbs or hind limbs, has been documented. A time-sampling method was used to assess behavior, where the frequency of immobility, swimming, and struggling was noted at 5-s intervals throughout the test session (Yankelevitch-Yahav et al., 2015).

#### 2.6.4 Single-bottle saccharin preference test

Prior to the test, the total volume of water consumed by each mouse over a 24-h period was measured. On the test day, the water bottle was replaced with a single bottle containing a freshly prepared 0.1% saccharin solution stored at room temperature. The mice were provided access to the saccharin solution for 24 h. The total volume of saccharin solution consumed during the test period was determined by weighing the saccharin bottles before and after the test. Saccharin preference was calculated by comparing the total volume of saccharin solution consumed during the test period with the total volume of water consumed during the pre-test period.

### 2.7 RNA sequencing and analysis

Total RNA was extracted from the frontal cortex using the TRIzol reagent (Life Technologies, Carlsbad, CA, USA), followed by purification using DNase I (Thermo Fisher Scientific, USA). RNA integrity and concentration were assessed using a NanoDrop spectrophotometer (Thermo Fisher Scientific, USA). RNA sequencing was performed by Novogene Co. Ltd. (Tokyo, Japan). Sequencing libraries were prepared using the NEBNext Ultra II RNA Library Prep Kit (Illumina) in accordance with the manufacturer’s protocol. Clean reads were aligned to the *Mus musculus* reference genome (GRCm39, Ensembl release 111) using STAR 2.7.0a. Alignment metrics indicated mapping rates exceeding 85% for all samples.

Gene expression levels were quantified as fragments per kilobase of transcript per million mapped reads using RSEM 1.3.3. Differentially expressed genes (DEGs) were identified using DESeq2 package 1.42.0 in R 4.3.2. The DESeq2 workflow included normalization and statistical testing using Wald tests for pairwise comparisons. DEGs were filtered based on fold change > 1 and adjusted *p*-value < 0.05 for Wald tests. One outlier sample was identified during the analysis. Functional enrichment analyses of the DEGs were conducted using Metascape (v3.5.20240901) with a cut-off criterion of adjusted *p*-value < 0.05 for all groups. Venn diagrams and bar plots for enrichment analysis were generated using OECloud tools^1^ provided by OE Biotech Co., Ltd. To validate the reliability of the RNA-seq results, we performed RT-PCR on a subset of representative genes from key enriched pathways, using the same sample set. The expression trends observed by RT-qPCR were consistent with the RNA-seq data, supporting the robustness of the transcriptomic findings (Supplementary Figure S2). Previous studies have shown that high-quality RNA-seq datasets, when processed under stringent protocols, provide reliable and reproducible gene expression profiles without requiring validation of every individual gene (Everaert et al., 2017).

Body temperature recordings, EEG/EMG measurements, and a subset of behavioral tests (open field test, object location test, and novel object recognition test) were performed using separate cohorts of mice. Although these cohorts were not used for molecular or transcriptomic analyses, all animals were subjected to the same siesta disruption protocols and housed under identical environmental conditions. This approach was used to avoid repeated invasive procedures in the same animals and to ensure data quality across different experimental modalities.

### 2.8 Statistical analysis

Statistical analyses were performed using SPSS version 29.0.0.0 (IBM Corp., Armonk, NY) and graphical representations were created using GraphPad Prism version 10.1.0 (GraphPad Software, San Diego, CA, USA). Normality of the data was assessed using the Shapiro-Wilk test, and homogeneity of variance was evaluated with Levene’s Test or an F-test, as appropriate. For parametric data, two-way ANOVA followed by Bonferroni’s post-hoc test was used for multiple comparisons, and unpaired two-tailed t-tests were applied for pairwise comparisons. Welch’s correction was applied when the assumption of equal variances was violated, as automatically determined by SPSS. Non-parametric data were analyzed using the Kruskal-Wallis test with Dunn’s post-hoc test. Data are presented as mean ± standard error of the mean.

For the statistical analysis of 24 h body temperature, activity, and sleep-related time-series data, which did not pass the equal variance tests, differences between two groups at specific time points were assessed using unpaired two-tailed t-tests. Statistical significance was set at *p* < 0.05, with significance denoted as follows: *p < 0.05.

## 3 Results

### 3.1 Body weight, diet intake, and serum stress response

**Figure 2:**
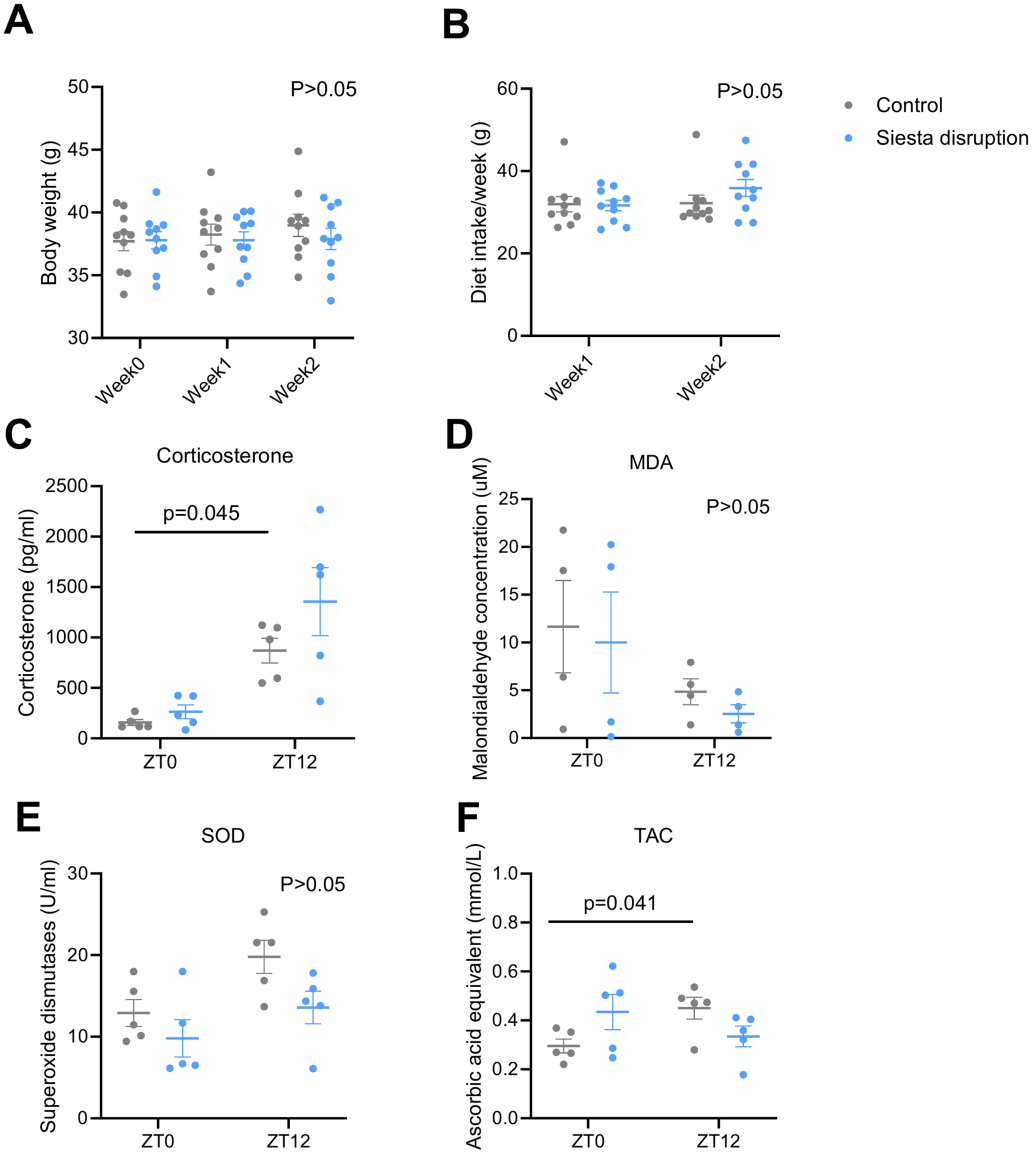
Effects of siesta disruption on body weight, diet intake, and serum stress markers. **(A,B)** Body weight and food intake remained stable across groups. *n* = 10 mice per group. **(C–F)** Serum levels of corticosterone, MDA, SOD, and TAC measured at ZT0 and ZT12. *n* = 4–5 mice per group. Data are presented as mean ± SEM. Statistical significance was assessed using two-way ANOVA with Bonferroni *post hoc* test or Kruskal–Wallis test followed by Dunn’s post hoc test with Bonferroni correction. Statistical significance was set at *p* < 0.05. Data are presented for the control (Con) group and the siesta disruption (Sd) group.

Figures 2A,B presents the results for body weight and dietary intake, which remained stable across experimental groups. No significant differences in serum corticosterone level (Figure 2C), MDA level (a marker of lipid peroxidation, Figure 2D), SOD activity (a key antioxidant enzyme (Michiels et al., 1994), Figure 2E), and TAC level (Figure 2F) were found between control and siesta disruption. In the control group, corticosterone level (*Z* = −2.67, *p* = 0.045) and TAC level (*F* (interaction) = 0.02, *p* = 0.041) were significantly higher at ZT12 than ZT0, indicating a time-dependent change, while, siesta disruption became flat and did not show any time-dependent change.

### 3.2 Transient arousal-related increase in body temperature and activity during ZT20–24

**Figure 3:**
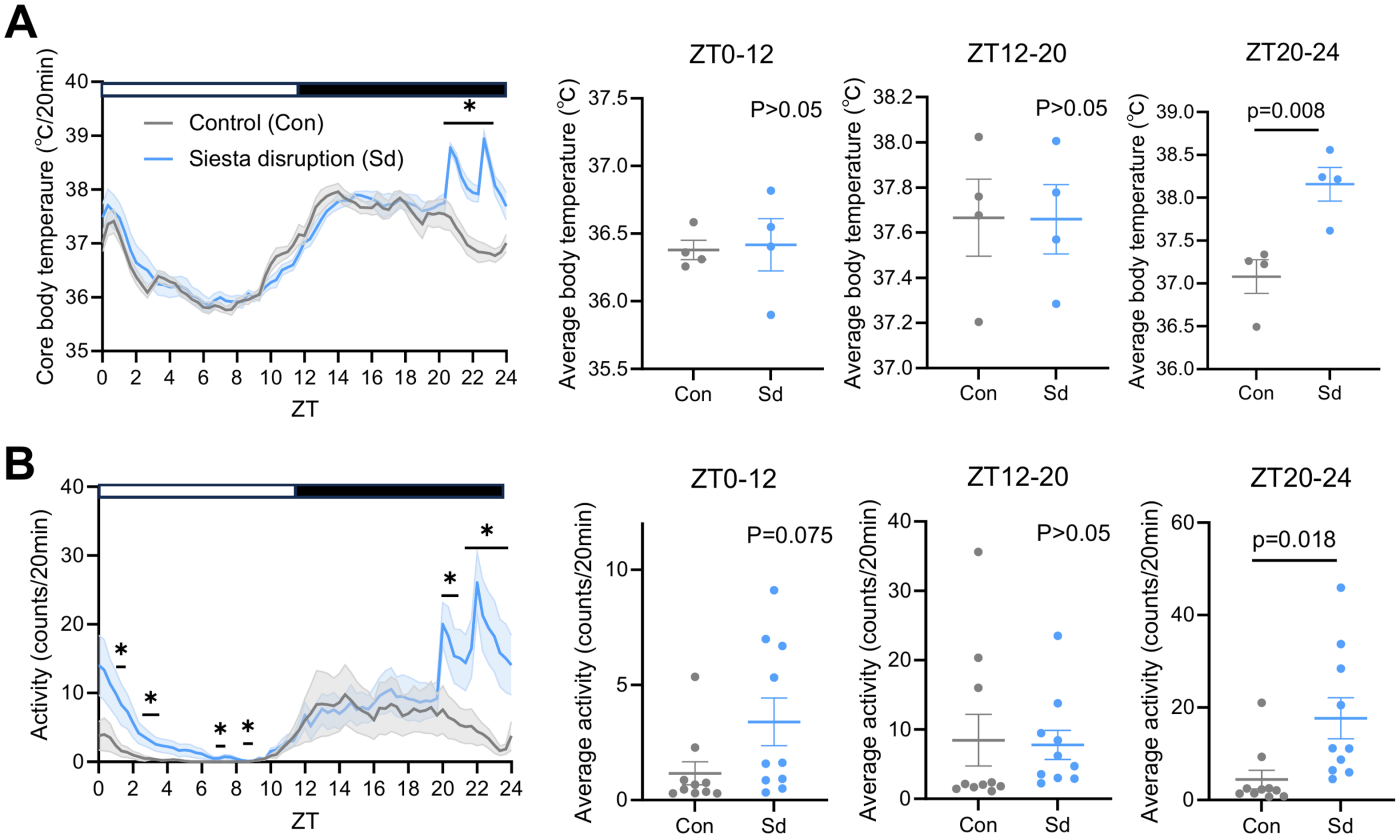
Effects of siesta disruption on body temperature and locomotor activity **(A)** Core body temperature over 24 h, along with the average body temperature during ZT0–12, ZT12-20 and ZT20-24. *n* = 4 mice per group. **(B)** Locomotor activity levels over 24 h, along with the average locomotor activity during ZT0–12, ZT12-20, and ZT20-24. *n* = 10 mice per group. Data are presented as mean ± SEM. Statistical significance was assessed using two-tailed unpaired t-tests. Statistical significance was set at *p* < 0.05. *p < 0.05 vs. control. Data are presented for the Con and Sd groups.

Both core body temperature and locomotor activity exhibited clear circadian rhythms, with lower levels during the light phase and higher levels during the dark phase. During the second half of the dark phase, both parameters declined, corresponding to the siesta period. To disrupt this rest phase, we introduced siesta disruption interventions at ZT20 and ZT22. A single intervention at ZT20 induced a brief increase in arousal, but its effect diminished quickly, allowing a rebound siesta around ZT22–23 (Supplementary Figure S1). A second intervention at ZT22 reactivated both physiological measures. These observations demonstrate that dual interventions are required to sustain arousal throughout the siesta window, as confirmed by increased body temperature (*F* = 0.02, *p* = 0.008) and locomotor activity (*F* = 7.82, *p* = 0.018) during ZT20-24 (Figures 3A,B), with notable elevations at specific time points detailed in Supplementary Table S1. During the light phase sleep (ZT0–12) and early dark phase (ZT12–20), core body temperature did not differ significantly between groups. However, locomotor activity was significantly elevated in the siesta disruption group at some special time points during the light phase: ZT1.67 (*F* = 26.22, *p* = 0.046), ZT2.67 (*F* = 16.42, *p* = 0.046), ZT3 (*F* = 28.14, *p* = 0.049), ZT7.33 (*F* = 23.33, *p* = 0.048), ZT8.67 (*F* = 8.32, *p* = 0.037), suggesting that the arousal effects of siesta disruption persist into the subsequent rest period.

### 3.3 Light-phase sleep architecture is preserved despite arousal changes

**Figure 4:**
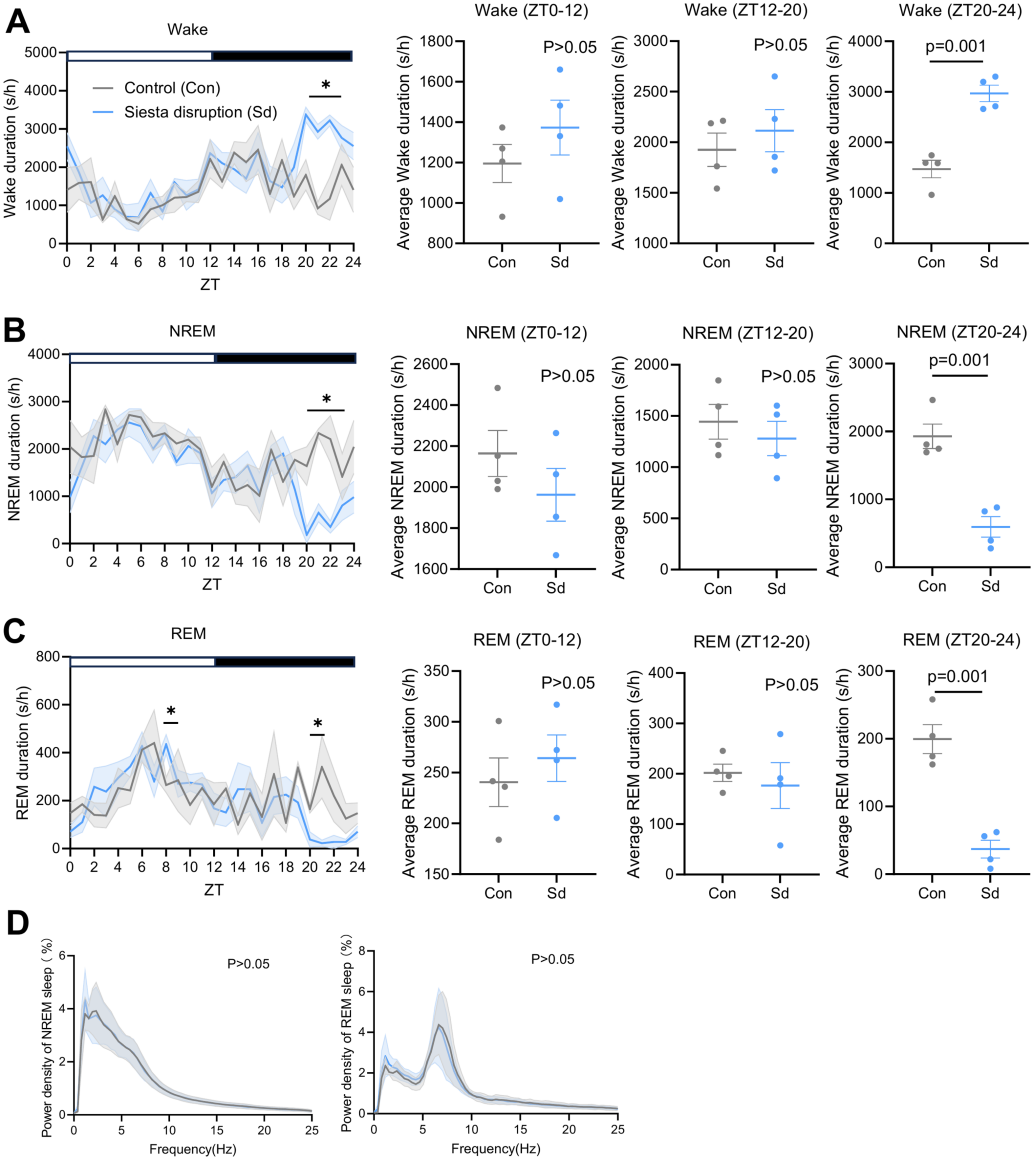
Effects of siesta disruption on sleep parameters. **(A–C)** Durations of wake, NREM sleep, and REM sleep across 24 h, and averaged within ZT0–12, ZT12–20, and ZT20–24. **(D)** Power density of NREM and REM sleep during light-phase sleep. *n* = 4 mice per group. Statistical significance was assessed using two-tailed unpaired t-tests. Statistical significance was set at *p* < 0.05. *p < 0.05 vs. control. Data are presented for the Con and Sd groups.

Interventions at ZT20 and ZT22 significantly increased wakefulness during ZT20–24 (*F* = 0.07, *p* = 0.001), particularly at ZT20 (*F* = 1.17, *p* = 0.012), ZT21 (*F* = 0.02, *p* = 0.000), and ZT22 (*F* = 18.54, *p* = 0.031) (Figure 4A), accompanied by a corresponding reduction in NREM sleep during the same period (*F* = 0.01, *p* = 0.001), especially at ZT20 (*F* = 1.83, *p* = 0.016), ZT21 (*F* = 1.25, *p* = 0.000), and ZT22 (*F* = 7.60, *p* = 0.029) (Figure 4B). REM sleep also decreased significantly across ZT20–24 (*F* = 0.66, *p* = 0.001), particularly at ZT20 (*F* = 1.54, *p* = 0.011) (Figure 4C). Overall, this protocol resulted in an approximate 70% reduction in total sleep during the intervention window, confirming the effectiveness of the siesta disruption model. Moreover, a significant rebound increase in REM sleep was observed in the siesta disruption group at ZT8 (*F* = 0.09, *p* = 0.015). In contrast, no significant differences were observed in the total durations of wakefulness, NREM sleep, or REM sleep during ZT0–12 and ZT12–20 among the groups (all *p* > 0.05). Furthermore, the power density of NREM and REM sleep during ZT0–12 was not affected by siesta disruption (Figure 4D). Although NREM delta power is a well-established marker of sleep pressure, no group differences were found, potentially due to rapid homeostatic compensation or the relatively limited contribution of siesta sleep to overall sleep drive. These results indicate that siesta disruption primarily alters sleep architecture during the intervention window (ZT20–24), while leaving the overall sleep patterns during the subsequent light phase (ZT0–12) largely unchanged.

### 3.4 Minimal behavioral impact of siesta disruption

**Figure 5:**
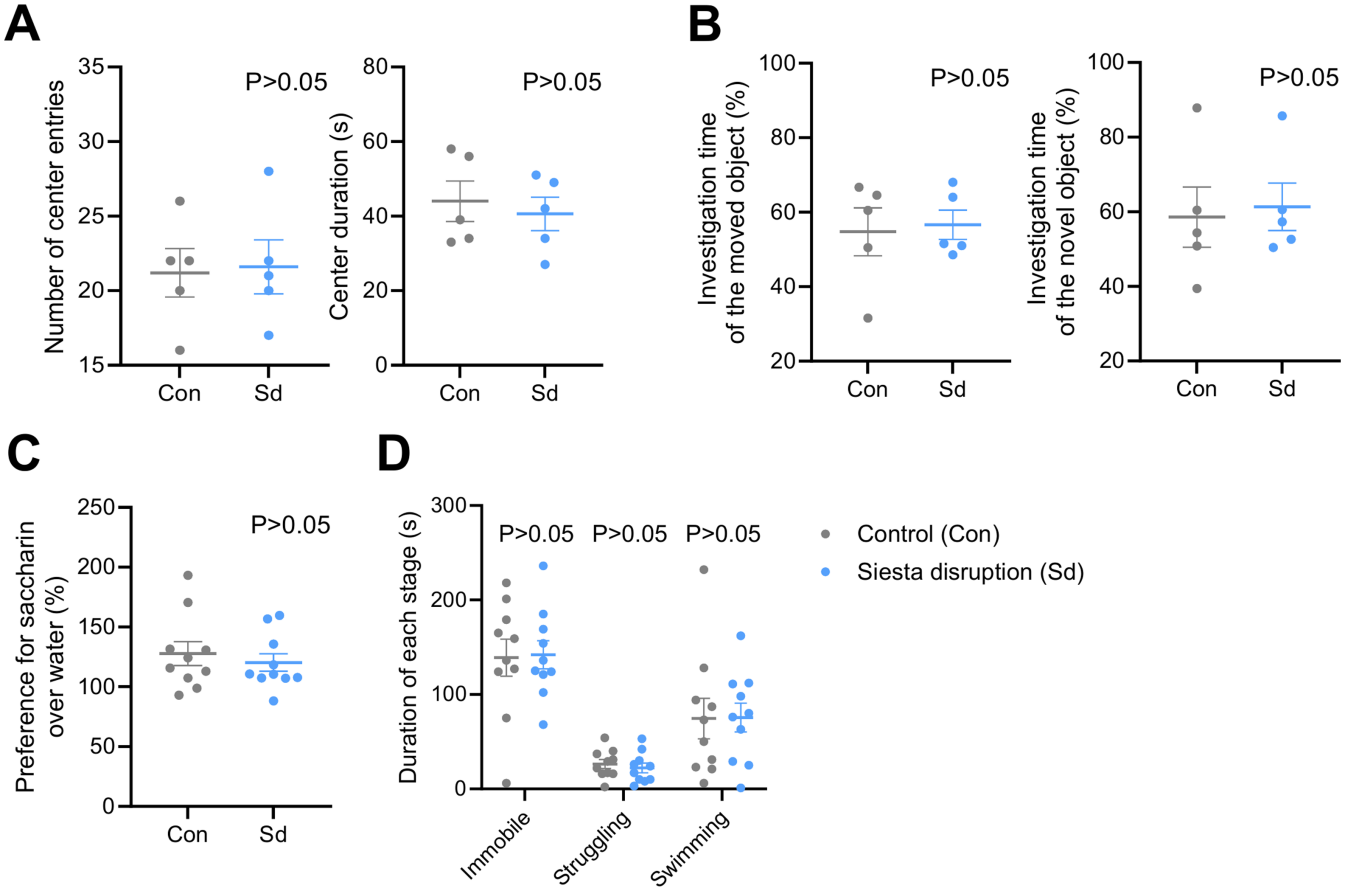
Effects of siesta disruption on anxiety-like, cognitive, hedonic, and depressive-like behaviors. **(A)** Open field test results showing the number of center entries and the time spent in the center zone. *n* = 5 mice per group. **(B)** Object recognition performance in the object location and novel object recognition tests, measuring exploration of moved and novel objects. *n* = 5 mice per group. **(C)** Saccharin preference test results assessing anhedonia-like behavior. *n* = 10 mice per group. **(D)** Forced swim test results evaluating immobility, swimming, and struggling durations. *n* = 10 mice per group. Data are presented as mean ± SEM. Statistical significance was assessed using two-tailed unpaired t-tests. *p* < 0.05 was considered statistically significant. No statistically significant differences were observed. Data are presented for the Con and Sd groups.

Behavioral responses to siesta disruption were assessed at the end of light phase using multiple paradigms. Anxiety-like behavior was evaluated using the open field test, which revealed no significant differences in the number of center entries or time spent in the center zone across the groups (Figure 5A). This result indicated that siesta disruption did not alter anxiety-like behavior. Cognitive performance was assessed using the object recognition test, which included the recognition of moved and novel objects. Both measures remained unaffected by siesta disruption (Figure 5B), indicating that cognitive performance remained intact. Hedonic behavior was assessed by measuring the preference for saccharin over water, and no differences were observed between the groups (Figure 5C), suggesting that hedonic behavior was not affected by siesta disruption. Finally, depression-like behavior was evaluated using the forced swim test, in which immobility, struggling, and swimming duration were similar between the groups (Figure 5D). While these results suggest minimal behavioral consequences of siesta disruption, caution is warranted in interpretation. The relatively small sample sizes may have limited the statistical power to detect subtle effects, and the paradigms used may not fully capture the range of possible functional alterations. These limitations should be considered when evaluating the absence of group differences. However, as our data showed no apparent trend toward behavioral differences between groups, we consider the lack of significance to reflect a true absence of effect, rather than a false negative.

### 3.5 Siesta disruption induced broad transcriptional engagement at ZT0 and ZT12

**Figure 6:**
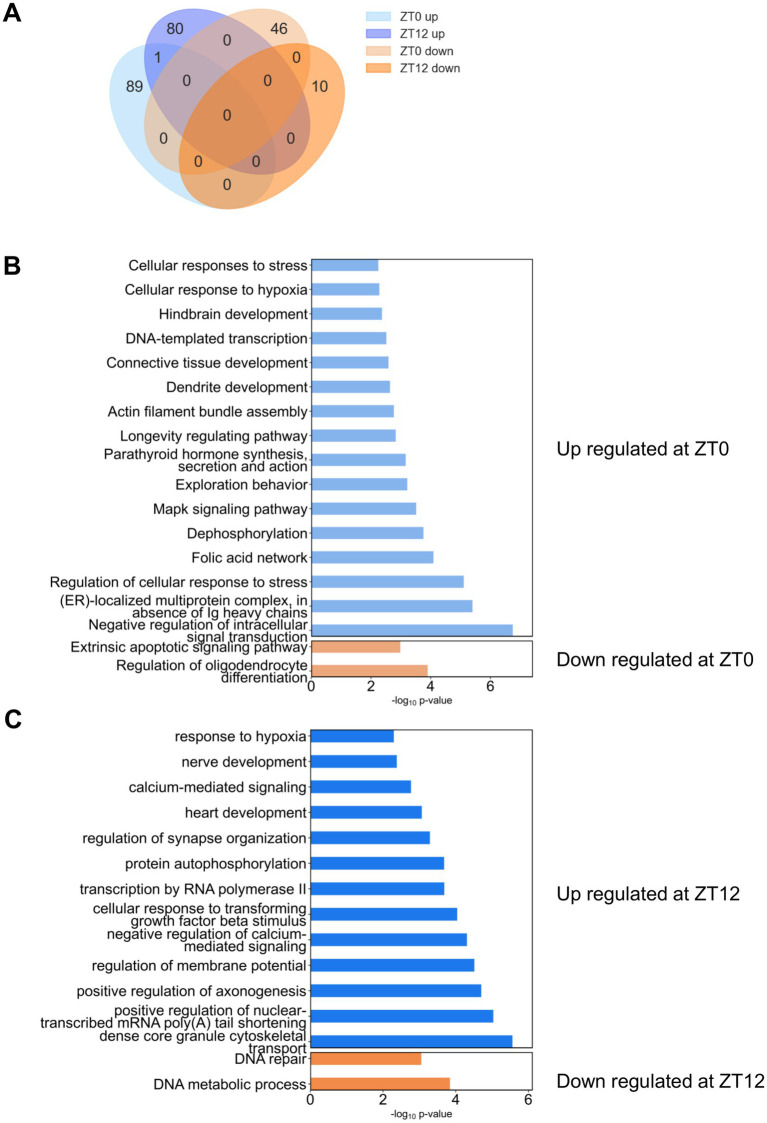
Transcriptional responses to siesta disruption in the cortex at ZT0 and ZT12. **(A)** Venn diagram showing the overlap and unique sets of differentially expressed genes (DEGs), including both upregulated and downregulated genes, in each siesta disruption group compared to the control group. *n* = 4–5 mice per group. **(B)** Functional enrichment analysis of DEGs at ZT0. Enriched terms for upregulated genes are shown in light blue; those for downregulated genes are shown in light orange. **(C)** Functional enrichment analysis of DEGs at ZT12. Enriched terms for upregulated genes are shown in blue; those for downregulated genes are shown in orange. Data are based on Metascape enrichment analysis of DEGs (adjusted *p* < 0.05). No additional statistical tests were performed beyond the enrichment analysis.

The Venn diagram (Figure 6A) reveals both overlapping and unique sets of DEGs in the siesta disruption groups compared to controls. At ZT0, 90 genes were upregulated and 46 were downregulated, while at ZT12, 81 genes were upregulated and 10 were downregulated. The complete lists of DEGs are provided in Supplementary Table S2. Notably, Pseudopodium-enriched atypical kinase 1 (*Peak1*) was the only gene consistently upregulated at both ZT0 and ZT12 (Supplementary Figure S3).

#### 3.5.1 ZT0: acute stress response and early cellular adaptation

Enrichment analysis was independently performed for the 90 up-regulated and 46 down-regulated genes at ZT0. All statistically enriched terms (based on –log₁₀ *p* values) are presented in Figure 6B. Terms derived from up-regulated genes are shown in light blue, while those from down-regulated genes are shown in light orange. Negative regulation of intracellular signal transduction, ER-localized multiprotein complex formation in the absence of immunoglobulin heavy chains, and Regulation of the cellular response to stress were the most prominent. These were followed by pathways associated with Dephosphorylation, MAPK signaling, Cellular response to hypoxia, and General cellular stress responses.

**Figure 7:**
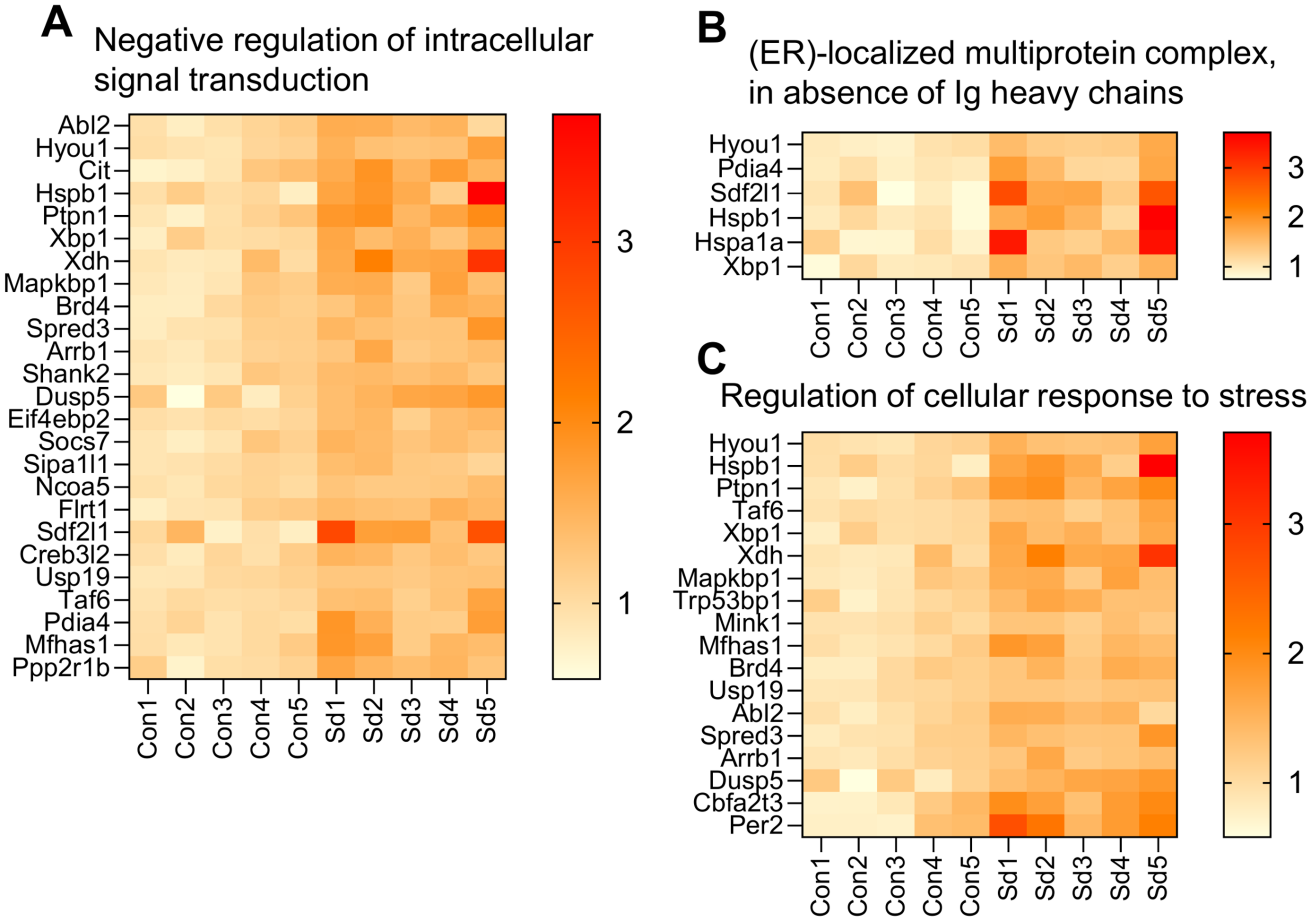
Heatmap showing expression levels of genes enriched in key biological processes at ZT0. *n* = 5 mice per group. **(A)** Genes involved in the negative regulation of intracellular signal transduction. **(B)** Genes associated with ER-localized multiprotein complexes in the absence of immunoglobulin heavy chains. **(C)** Genes related to the regulation of cellular response to stress. Data are presented for the Con and Sd groups.

Specifically, the term “Negative regulation of intracellular signal transduction” included 25 enriched genes, such as Dual specificity phosphatase 5 (*Dusp5*) and Suppressor of cytokine signaling 7 (*Socs7*) (Figure 7A). For the term “ER-localized multiprotein complex, in the absence of Ig heavy chains” six genes were enriched, including X-box binding protein 1 (*Xbp1*) and Protein disulfide isomerase family A member 4 (*Pdia4*) (Figure 7B). The term “Regulation of cellular response to stress” included 12 enriched genes, such as *Xbp1* and Heat shock protein beta-1 (*Hspb1*) (Figure 7C).

#### 3.5.2 ZT12: synaptic remodeling and structural adaptation

Enrichment analysis was independently performed for the 81 up-regulated and 10 down-regulated genes at ZT12 (Figure 6C). Terms derived from up-regulated genes are shown in blue, while those from down-regulated genes are shown in orange. Dense core granule cytoskeletal transport, Positive regulation of nuclear-transcribed mRNA poly(A) tail shortening, Positive regulation of axonogenesis, Regulation of membrane potential, Negative regulation of calcium-mediated signaling, and Cellular response to transforming growth factor beta stimulus were the most prominently enriched terms. These pathways are primarily associated with synaptic remodeling and structural adaptation.

**Figure 8:**
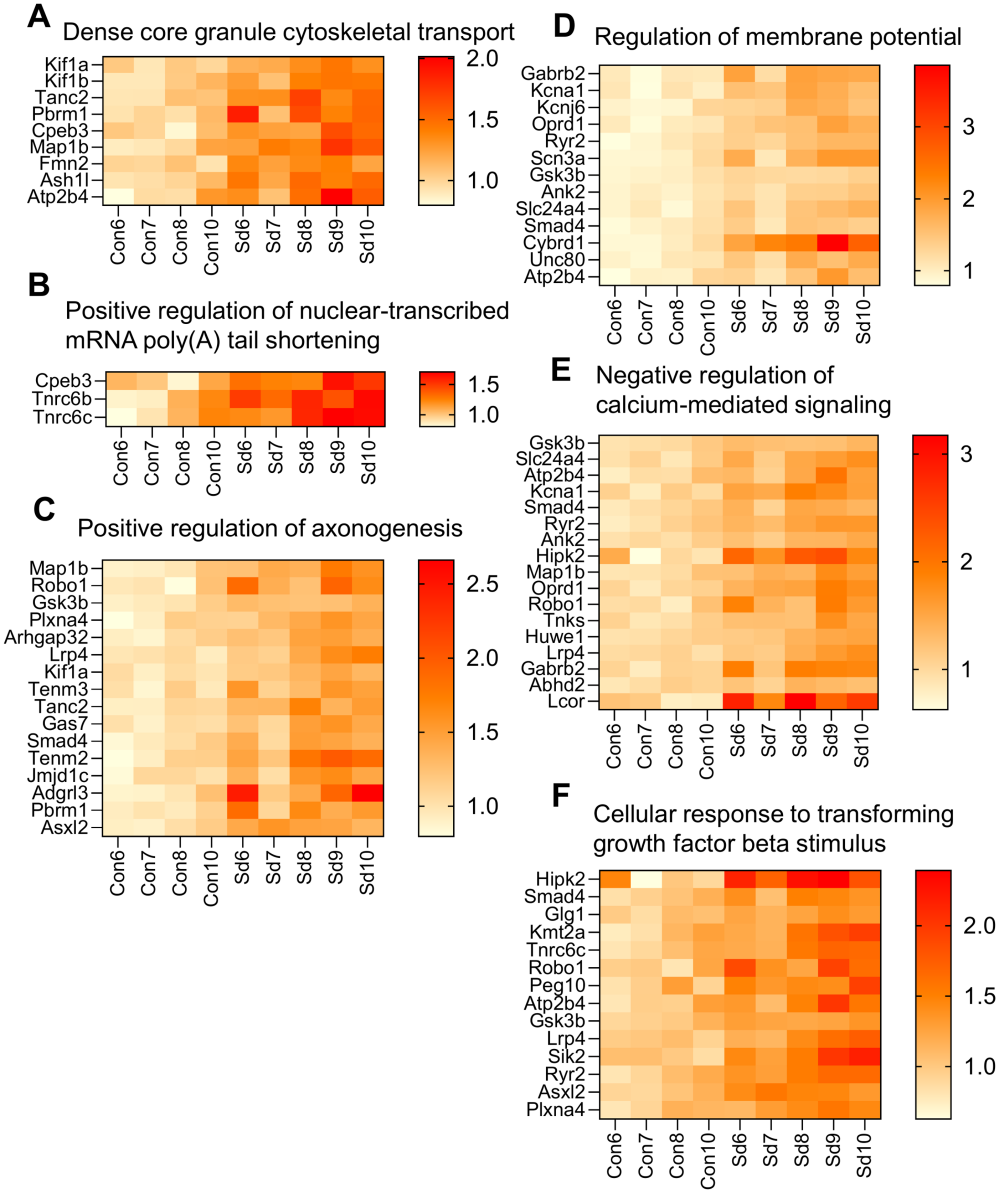
Heatmap showing expression levels of genes enriched in key biological processes at ZT12. *n* = 4–5 mice per group. **(A)** Genes related to dense core granule cytoskeletal transport. **(B)** Genes involved in the positive regulation of nuclear-transcribed mRNA poly(A) tail shortening. **(C)** Genes involved in the positive regulation of axonogenesis. **(D)** Genes involved in the regulation of membrane potential. **(E)** Genes involved in the negative regulation of calcium-mediated signaling. **(F)** Genes involved in the cellular response to transforming growth factor beta stimulus. Data are presented for the Con and Sd groups.

Specifically, the term “Dense core granule cytoskeletal transport” included 9 enriched genes, such as Kinesin family member 1A (*Kif1a*) (Figure 8A). For the term “Positive regulation of nuclear-transcribed mRNA poly(A) tail shortening” three genes were enriched, including Cytoplasmic polyadenylation element binding protein 3 (*Cpeb3*) (Figure 8B). The term “Positive regulation of axonogenesis” included 16 enriched genes, such as Microtubule-associated protein 1B (*Map1b*), Kinesin family member 1A (*Kif1a*), and Roundabout guidance receptor 1 (*Robo1*) (Figure 8C). The term “Regulation of membrane potential” included 13 enriched genes, such as Potassium voltage-gated channel subfamily A member 1 (*Kcna1*), Potassium inwardly-rectifying channel subfamily J member 6 (*Kcnj6*), Sodium channel voltage-gated type III alpha subunit (*Scn3a*), and ATPase plasma membrane calcium transporting 4 (*Atp2b4*) (Figure 8D). For the term “Negative regulation of calcium-mediated signaling,” 17 genes were enriched, including *Atp2b4* (Figure 8E). The term “Cellular response to transforming growth factor beta stimulus” included 14 enriched genes, such as SMAD family member 4 (*Smad4*) (Figure 8F).

## 4 Discussion

To the best of our knowledge, this study is the first to comprehensively investigate the effects of siesta disruption, with a particular focus on time-specific transcriptional responses in the mouse frontal cortex. Our findings demonstrate that siesta disruption induced by bedding changes has a relatively modest overall impact, with no significant alterations observed in systemic stress levels, light-phase sleep, or behavioral performance.

Our previous data showed that cage changes for four times every 30 min between ZT4-6 increased locomotor activity and suppressed sleep without affecting peripheral clock gene expression (Tahara et al., 2015). Although the current study employed bedding change instead of full cage replacement, the principle remains similar—mild, non-invasive stimulation during rest periods that preserves systemic circadian rhythm integrity. Analysis of body temperature and locomotor activity revealed no shifts in circadian phase onset, although activity data indicated that the arousal effects of siesta disruption extended into the subsequent light phase rest period. Furthermore, enrichment analysis did not reveal significant alterations in circadian-related gene pathways, supporting the notion that our intervention disrupts siesta-specific sleep regulation without inducing broader circadian misalignment. These findings support the potential of bedding change as a mild and feasible approach for inducing siesta disruption in rodents.

The gene enrichment profile observed at ZT0 suggests that the cortex responds to acute stress through the rapid activation of intracellular signaling inhibitors and Endoplasmic reticulum (ER)-associated adaptive mechanisms. Notably, the enrichment of terms such as Negative regulation of intracellular signal transduction and Regulation of cellular response to stress highlights an early compensatory response to external stimuli. Key regulators such as *Dusp5* and *Socs7* are known to downregulate MAPK and JAK/STAT signaling pathways, respectively, serving as critical modulators of excessive stress signaling and helping to preserve cellular homeostasis (Kucharska et al., 2009; Cooney, 2002). The enrichment of *Xbp1* and *Pdia4* under the term ER-localized multiprotein complex (in the absence of Ig heavy chains) implies that ER stress responses are also activated at this early time point. *Xbp1* functions as a master transcription factor of the unfolded protein response, promoting ER-associated degradation and the restoration of protein folding capacity (Jiang et al., 2015). The co-enrichment of *Pdia4*, a protein disulfide isomerase that facilitates proper protein folding, supports the view that ZT0 reflects a phase of early ER stress response, likely contributing to cellular recovery from protein damage or misfolding (Liu et al., 2025). In addition, the involvement of *Hspb1* in regulation of cellular response to stress suggests engagement of cytoprotective chaperone systems. *Hspb1* is known to stabilize cytoskeletal components and suppress apoptotic signaling, further contributing to stress tolerance and cytoskeletal integrity (Clarke and Mearow, 2013). Together, these findings indicate that ZT0 represents a time window of heightened cellular vigilance and rapid stress adaptation, orchestrated through negative feedback mechanisms in signal transduction, ER proteostasis, and molecular chaperone activity.

In contrast, the findings at ZT12 highlight the enrichment of several key biological processes, including synaptic remodeling and structural adaptation. These processes are critical for maintaining neuronal plasticity and functionality during the active phase. Enriched terms such as “Dense core granule cytoskeletal transport,” “Positive regulation of axonogenesis,” and “Regulation of membrane potential” suggest active processes involved in synaptic rearrangement and ion homeostasis, both of which are essential for proper neuronal communication.

The role of *Kif1a*, enriched in “Dense core granule cytoskeletal transport” underscores its importance in axonal transport, which is crucial for synaptic vesicle trafficking and neuronal function (Chiba et al., 2023). Furthermore, the enrichment of *Cpeb3* in “Positive regulation of nuclear-transcribed mRNA poly(A) tail shortening” highlights its role in mRNA regulation at synaptic sites, impacting synaptic plasticity (Huang et al., 2006). Together, these genes emphasize the dynamic nature of synaptic regulation, with both protein transport and translational control contributing to synaptic remodeling. The term “Positive regulation of axonogenesis” enriched with *Map1b* and *Robo1*, suggests active mechanisms of axon growth and guidance. *Map1b* regulates microtubule dynamics critical for axon elongation (Ziak et al., 2024), while *Robo1* mediates axonal guidance and synaptic positioning (López-Bendito et al., 2007). These findings are consistent with known mechanisms of neural circuit formation during synaptic remodeling.

Ion channel regulation, highlighted by genes such as *Kcna1* and *Atp2b4* in “Regulation of membrane potential” points to the importance of maintaining cellular excitability. *Kcna1*, a potassium channel, plays a vital role in neuronal repolarization (Ni et al., 2024), while *Atp2b4* regulates calcium efflux, ensuring calcium homeostasis during synaptic transmission (Huang et al., 2010). Furthermore, the involvement of *Smad4* in “Cellular response to transforming growth factor beta stimulus” suggests that *Smad4*, as a central mediator of transforming growth factor beta signaling—which is known to regulate synaptic plasticity—may contribute to the adaptive molecular response observed at ZT12 (Zhou et al., 2003). In summary, the enrichment of genes related to cytoskeletal transport, axonogenesis, ion channel regulation, and calcium signaling at ZT12 reflects a coordinated response involved in synaptic remodeling and structural adaptation.

Among the differentially expressed genes, *Peak1* was uniquely upregulated at both ZT0 and ZT12, with its correlated genes showing distinct pathway enrichment at each time point. At ZT0, *Peak1*-associated genes were mainly enriched in “Regulation of response to endoplasmic reticulum stress” (Supplementary Figure S3B), whereas at ZT12, they were predominantly linked to “Positive regulation of axon extension” and “Organelle transport along microtubules” (Supplementary Figure S3C). Although these associations are correlative, they are consistent with findings in cancer models, where *Peak1*—a non-receptor tyrosine kinase associated with the cytoskeleton—mediates signaling crosstalk between transforming growth factor beta receptors and integrin/Src/MAPK pathways (Agajanian et al., 2015; Wang et al., 2010). The recurrence of these pathways in our data suggests that *Peak1* may function as a regulatory node involved in both stress responses and structural remodeling under siesta disruption. While its role in the brain remains largely uncharacterized, these results extend the potential relevance of *Peak1* beyond cancer biology and point to its possible involvement in neuronal adaptation to sleep-related stress.

In summary, siesta disruption induced modest but time-specific changes in the mouse frontal cortex. While systemic markers of stress remained relatively stable, cortical transcriptomes revealed distinct phase-dependent responses: stress-adaptive pathways dominated at ZT0, whereas synaptic remodeling programs emerged at ZT12. These findings underscore the importance of siesta sleep in supporting cortical homeostasis and highlight circadian timing as a key modulator of stress adaptation and neuronal plasticity. Notably, *Peak1* was the only gene consistently upregulated at both time points, and its associated pathways—ER stress at ZT0 and cytoskeletal remodeling at ZT12—are consistent with findings from non-neuronal systems, suggesting a broader role for *Peak1* in cellular adaptation. Given that only two time points were examined, our data provide a snapshot rather than a full circadian profile. Future studies should expand temporal resolution and integrate multi-omics analyses across brain regions to clarify the dynamic roles of siesta sleep in neural regulation. In addition, broader and more targeted behavioral assessments are required to fully elucidate the functional consequences of siesta disruption.

## Data Availability

The datasets presented in this study can be found in online repositories. The names of the repository/repositories and accession number(s) can be found at: https://www.ncbi.nlm.nih.gov/, PRJNA1219326.

## Footnotes

^1^ https://cloud.oebiotech.com
