# Supplementary material for: Time-dependent cortical responses to siesta disruption in male mice
Source: Front Neurosci. 2025 Aug 22;19:1613747. doi: 10.3389/fnins.2025.1613747 (PMC12411512; doi:10.3389/fnins.2025.1613747)
Supplement: Supplementary file 1 [file Data_Sheet_1.pdf]

Supplementary Methods (for Supplementary Figure S2)

RT-qPCR validation of ER stress–related genes

Total RNA was extracted from the frontal cortex using the same protocol as for RNA-seq. Real-time reverse transcription PCR (RT-qPCR) was performed using the One-Step TB Green PrimeScript RT-PCR Kit (Takara Bio Inc., Shiga, Japan). PCR was conducted using a Thermal Cycler Dice Real-Time systemIII (Takara Bio Inc., Shiga, Japan). The primers used for Gapdh and target genes are listed below:

glyceraldehyde-3-phosphate dehydrogenase (*Gapdh*):
forward: 5′-TGGTGAAGGTCGGTGTGAAC-3′,
reverse: 5′-AATGAAGGGGTCGTTGATGG-3′;
immunoglobulin binding protein (*Bip*):
forward: 5′-TGTCTTCTCAGCATCAAGCAAGG-3′,
reverse: 5′-CCAACACTTCCTGGACAGGCTT-3′;
X-box binding protein 1 (*Xbp1*):
forward: 5′-TGGACTCTGACACTGTTGCCTC-3′,
reverse: 5′-TAGACCTCTGGGAGTTCCTCCA-3′;
protein disulfide isomerase 4 (*Pdia4*):
forward: 5′-TGGGCTCTTTCAGGGAGATGGT-3′,
reverse: 5′-GGGAGACTTTCAGGAACTTGGC-3′; and
heat shock protein beta-11 (*Hspb1*):
forward: 5′-GCTCACAGTGAAGACCAAGGAAG-3′,
reverse: 5′-TGAAGCACCGAGAGATGTAGCC-3′.

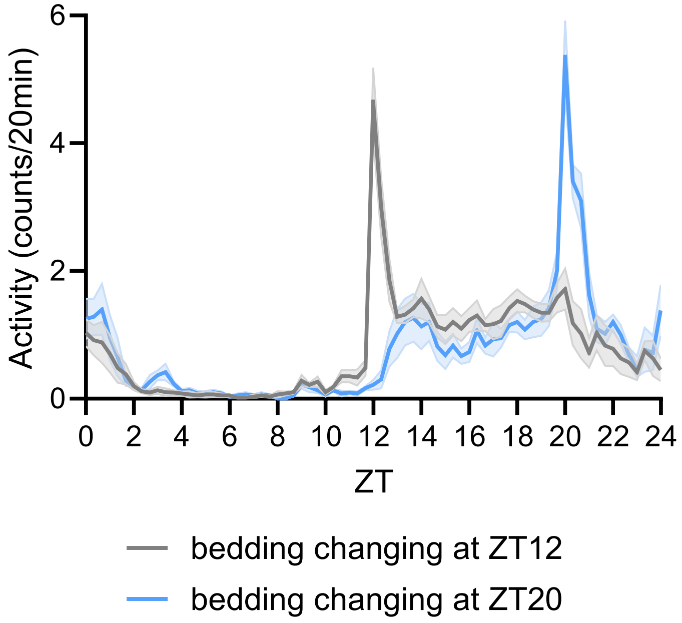

Supplementary Figure S1. Effects of a single bedding change at ZT12 or ZT20 on locomotor activity. n = 6 mice per group. This figure represents a separate experimental cohort designed to examine the arousal response induced by a single bedding change at different time points. The intervention was applied either at ZT12 (onset of the dark phase) or ZT20 (late dark phase), and locomotor activity was monitored thereafter.

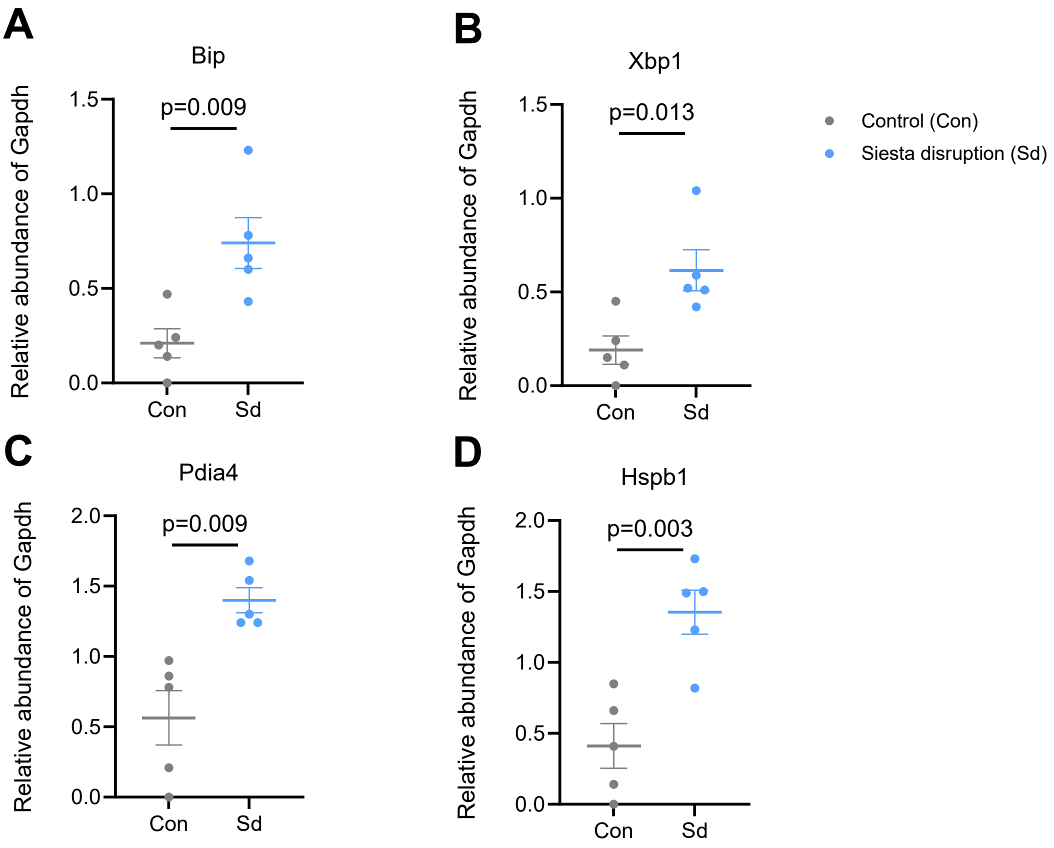

Supplementary Figure S2. Validation of ER stress-related gene expression at ZT0 using RT-PCR. Relative mRNA abundance of (A) *Bip*, (B) *Xbp1*, (C) *Pdia4*, and (D) *Hspb1* in the frontal cortex of control (Con) and siesta disruption (Sd) groups ($n = 5$ per group). Expression levels were normalized to *Gapdh* and analyzed using the $2^{-\Delta\Delta Ct}$ method. Each dot represents an individual animal; error bars denote mean ± SEM. Statistical comparisons were performed using unpaired two-tailed t-tests. Notably, while *Bip*—a key ER stress marker—was not significantly detected in RNA-seq, it showed robust upregulation in RT-PCR, highlighting the enhanced sensitivity of gene-specific validation.

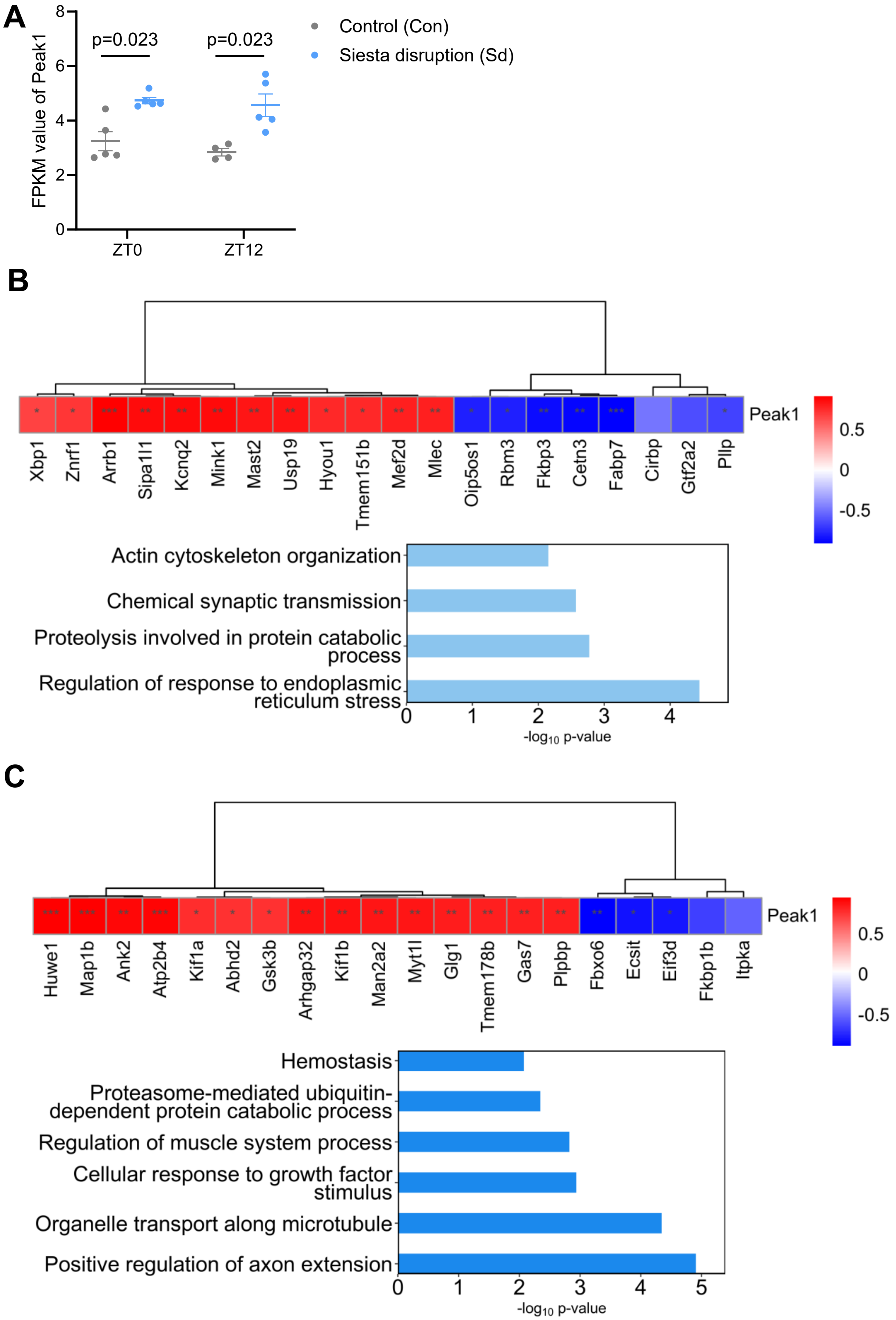

Supplementary Figure S3

Supplementary Figure S3. *Peak1* correlation networks and pathway enrichment. n = 4-5 mice per group. (The intensity of the color indicates the strength of the correlation: deeper red represents a stronger positive correlation, while deeper blue indicates a stronger negative correlation.)

(A) Expression levels of *Peak1* across experimental conditions and time points. Statistical testing was performed using Wald tests within the DESeq2 pipeline. (B) Heatmap showing genes significantly correlated with *Peak1* at ZT0 and Metascape enrichment analysis revealed that *Peak1*-correlated genes at ZT0 were mainly associated with "ER stress-related pathways". (C) Heatmap showing genes significantly correlated with *Peak1* at ZT12 and Metascape enrichment analysis revealed that Peak1-correlated genes at ZT12 were mainly associated with "Positive regulation of axon extension" and "Organelle transport along microtubules". Statistical significance was assessed using Spearman correlation between *Peak1* and other DEGs. $^*p < 0.05$, $^{**}p < 0.01$, $^{***}p < 0.001$. Spearman correlation and cluster heatmap were generated using OECloud tools available at https://cloud.oebiotech.com. Data are presented for the control (Con) group and the siesta disruption (Sd) group.

Time (ZT)	Core Body Temperature F-values	Core Body Tempture p-values	Locomotor Activity F-values	Locomotor Activity p-values
20			0.00	0.017
20.33			1.71	0.038
20.67	2.90	0.011		
21	0.86	0.007		
21.33	0.11	0.024		
21.67	0.01	0.027	3.59	0.034
22	0.00	0.022	6.50	0.001
22.33	0.47	0.023	12.84	0.009
22.67	0.18	0.001	16.34	0.013
23	0.64	0.001	18.09	0.014
23.33	0.62	0.005	24.39	0.018
23.67	0.95	0.027	22.79	0.027

Supplementary Table S1.
Summary of statistical comparisons for core body temperature and locomotor activity at each Zeitgeber time (ZT) point during the siesta disruption. F-values and p-values are reported for each significant time point. Empty cells indicate non-significant.

up regulated at ZT0		up regulated at ZT12		down regulated at ZT0	down regulated at ZT12
Pfkfb3	Creb3l2	Zbed6	Cpeb3	Baiap2l2	Fkbp1b
Mlec	Aff3	Ryr2	Smad4	Hmgb2	Kirrel2
Trmt61b	Sipa1l1	Fam120c	Smg1	Gm12371	Itpka
Gfod1	Kcnq2	Kcna1	Tnks	Gm56450	Rnaseh2b
Adcy3	Peak1	Tenm2	Qser1	Gm30455	Ecsit
Cdyl2	Nwd1	Tanc2	Huwe1	Opalin	Dnase2a
Kdm5c	Gm4949	Tex9	Tenm3	Pcolce2	C230037L18Rik
Slc36a1	Brd4	Gm37608	Peak1	Iqcb1	Eif3d
Per2	Sipa1l3	Tnrc6b	Spata31f1c	Cetn3	Rfc5
Ptpn1	Smim3	Gm57395	Gabrb2	Gtf3c6	Fbxo6
Hyou1	Klhl29	Adgrl3	Man2a2	Rbm3	
Gtf3c1	Gm37885	Myt1l	Plxna4	Rft1	
Mfhas1	Hif3a	Unc80	Nufip2	Nme7	
Fosb	Wipf2	Slc24a4	Cybrd1	Gm44678	
P4ha1	Tmem151b	Cep350	Kmt2e	Phtf1os	
Xbp1	Zfp236	Zfp398	Tmem178b	Lhfpl3	
Rin1	Rundc1	Sik2	B230209E15Rik	Mir124-2hg	
Samd4b	Taf6	Scn3a	Lrp4	Fabp7	
Eif4ebp2	Txnrd1	Hipk2	Gsk3b	Gpr34	
Glns-ps1	Synj2	Map1b	Jmjd1c	Haus2	
Gpr68	Ccdc88c	Cdk12	Slco5a1	Aspa	
Ppp2r1b	Znfx1	Pdzd2	Gas7	Gtf2a2	
Prdm2	Gm42656	Lcor	Pbrm1	Cntn6	
Mapkbp1	Ppm1h	Ash1l	Kif1a	Chrnb3	
Pdia4	Ago2	St8sia1	Tet2	Hmgb3	
Cbfa2t3	Cables1	Ank2	Abhd2	Itgav	
Kdm6b	Dusp5	Arhgap32	Pappa2	Tfrc	
Zbtb4	Flrt1	Ksr2	Fmn2	6330415G19Rik	
Arrb1	Hspb1	Kif1b	Gm17167	Myom2	
Sdf2l1	Shank2	Klf12	BC049715	Cirbp	
Gse1	Atmin	Peg10	Atp2b4	Fkbp3	
Mef2d	Mink1	Gm38020	Zkscan16	Igfbp5	
Xdh	Spred3	Gm1043	Tnrc6c	C730002L08Rik	
2700029L08Rik	Hspa1a	Nav1	Kmt2a	Aqp9	
Usp19	Ncoa5	Gm38077	Faxc	Samd3	
Zbtb40	Zfhx2	Plagl1		Hace1	
Tnfrsf25	Abl2	Plpbp		Oip5os1	
Slc25a25	Socs7	Zfp704		Glmn	
A730060N03Rik	Slc66a2	Amotl1		Gpr21	
Gm38190	Cbs	Tcf20		Klhdc9	
Cit	Dusp11	Oprd1		Il33	
Trp53bp1	Rasl11a	Sbno1		Creg1	
Mast2	Hif1an	Robo1		Suclg2	
Fam171a1	Hs6st3	Asxl2		9030407P20Rik	
Sgsm1		Glg1		Ccdc90b	
Znrf1		Kcnj6		Pllp	

Supplementary Table S2. Complete list of differentially expressed genes (DEGs) at ZT0 and ZT12. Genes are grouped by direction of change and time point (ZT0 upregulated, ZT0 downregulated, ZT12 upregulated, ZT12 downregulated, relative to control). DEGs were identified using the DESeq2 workflow, including normalization and statistical testing with Wald tests for pairwise comparisons. Genes were filtered based on a fold change > 1 and an adjusted p-value < 0.05.
